# Supplementary material for: Fermented Feed Supplement Relieves Caecal Microbiota Dysbiosis and Kidney Injury Caused by High-Protein Diet in the Development of Gosling Gout
Source: Animals (Basel). 2020 Nov 17;10(11):2139. doi: 10.3390/ani10112139 (PMC7698594; doi:10.3390/ani10112139)
Supplement: Supplementary file 1 [file animals-10-02139-s001.pdf]

Supporting information

Table S1. Effects of protein levels on Feed intake and BW in goslings (***Experiment one***).

Indexes	Diets				RMSE	*P*-value
	160CP	180CP	200CP	220CP		
N (♀:♂=1:1)	12	12	12	12	-	
Feed intake, g						
Day 6	41.3	41.2	40.3	41.3	-	-
Day 8	56.3	56.8	49.8	56.9	-	-
Day 10	79.2	83.1	84.5	83.1	-	-
Day 12	95.9	98.5	88.7	100.5	-	-
Day 14	98.3	88.1	70.7	94.1	-	-
BW, g						
Day 3	102.7	100.0	103.1	101.2	2.9	0.20
Day 10	241.3	278.0	259.5	289.8	25.6	0.58
Day 15	451.7	536.3	467.6	544.9	39.9	0.19

CP: crude protein, BW: body weight.

We observed the effects of high-protein diets on the feed intake in goslings and found that from the age of 12 d, the feed intake started to drop in high protein diet groups, whereas there was a minor increase in the control group (160CP). This might be attributed to the loss of appetite due to visceral gout that mostly occurred in goslings of age 10–15 d. Surely, because of the lack of repeats in feed intake measurements, these changes might come from the experimental errors, and more experiments were needed to explore the effects of high-protein diets on production performance in geese. Despite the differences in feed intake, the live weight of goslings was similar among the different protein level groups. This is in line with the findings in chickens (Guo *et al.*, 2005). A reason for no significant variation in body weight among the groups might be the rapid disease paroxysm before death in the short experiment period.

Table S2. Effects of high-protein diets and fermented feed on Feed intake and BW in goslings (***Experiment two***)

	CN		HP		*P* value
	CN	CN-F	HP	HP-F	
ADFI, g	76.82±0.91[b]	78.15±1.73[c]	70.98±1.30[a]	76.97±1.32[b]	0.006
BW_1, g	79.50±1.38	78.75±1.73	82.89±1.29	80.39±0.97	0.194
BW_{35}, g	1380.0±85.8[b]	1260.0±50.6[ab]	1183.3±25.8[a]	1333.3±47.6[ab]	0.107
ADG, g	37.16±2.45[b]	33.75±1.45[ab]	31.44±0.76[a]	35.80±1.38[ab]	0.104
F/G, g/g	2.11±0.16	2.33±0.12	2.26±0.05	2.16±0.08	0.486

[1]Values are the means of 6 replicates with 15 birds each.

[2] ADFI, average daily feed intake; BW, body weight; ADG, average daily gain; F/G, the ratio of feed to gain.

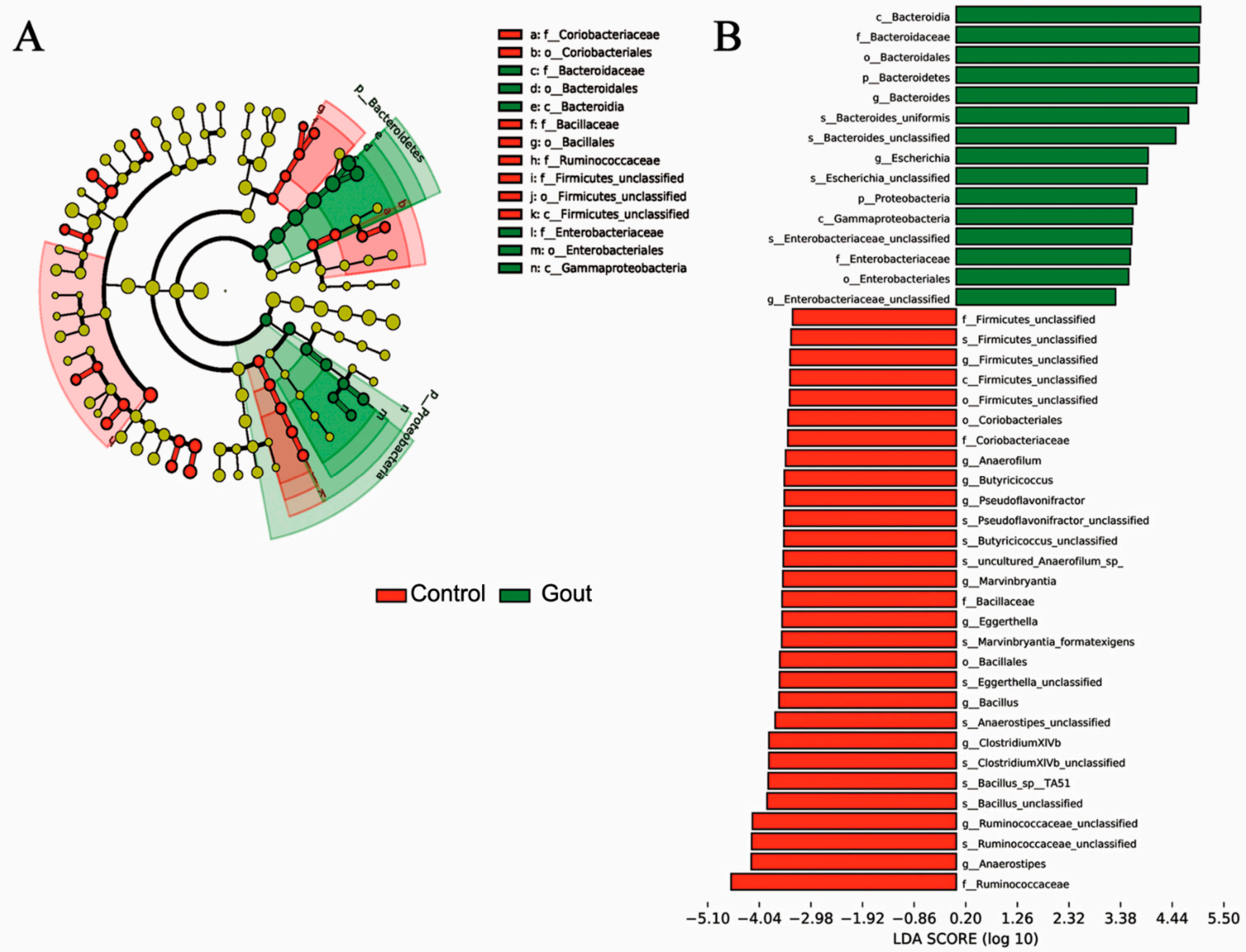

Figure S1. LefSe identified the most differential genera between the gout goslings and healthy controls. (A) cladogram; (B) histograms. Red and green represent healthy controls (from the 160CP group) and hyperuricaemia samples, respectively. **(*Experiment one*)**

The discrepant microbial species with a reduced significance threshold (LDA score > 2) between the gout goslings and healthy controls were also identified by the LefSe method (Fig. S1). The results showed that the phylum Bacteroidetes and its derivative (Bacteroidia, Bacteroidales, Bacteroidaceae, Bacteroides, and Bacteroides_unclassified), the phylum Proteobacteria and its derivatives (Enterobacteriales and Enterobacteriaceae) and the class Gammaproteobacteria were high in the intestinal microbiota of the gout goslings. On the contrary, the order Coriobacteriales and its derivative (Coriobacteriaceae), the order Bacillales and its derivative (Bacillaceae), the family Ruminococcaceae and some unclassified species in phylum Firmicutes were all high in the intestinal microbiota of healthy goslings.
